# Incidental discovery of a large mesenteric lipoma in a child following minor trauma: A case report

**DOI:** 10.1016/j.ijscr.2025.111636

**Published:** 2025-07-09

**Authors:** Abdelrahman S. Elnour, Abdelrahman S. Elnour, Alshazly Mohamed, Adel M. Osman, Mosab I. Ahmed

**Affiliations:** a Nyala Teaching Hospital, Sudan; b Sudan Medical Specialization Board, Sudan

**Keywords:** Mesenteric lipoma, Abdominal mass, Pediatric, Case report

## Abstract

**Introduction:**

Mesenteric lipomas are rare in children, with highly variable clinical presentations ranging from mild abdominal symptoms to acute bowel obstruction. Accurate imaging is essential for diagnosis, and early detection followed by complete surgical excision results in excellent outcomes with a low risk of recurrence.

**Case presentation:**

We report a 12-year-old female who presented with left leg laceration following a minor road traffic accident. While abdominal ultrasound performed as part of trauma evaluation revealed no acute injuries, it incidentally identified a well-defined intra-abdominal mass. Subsequent contrast-enhanced CT confirmed a homogeneous, intraabdominal low-attenuation lesion consistent with a lipoma. Elective laparotomy revealed a solitary, encapsulated tumor arising from the transverse colon mesentery, which was excised without bowel resection. Histopathology confirmed a mature lipoma. The postoperative course was uneventful, and no recurrence was observed after one year of follow-up.

**Discussion:**

Mesenteric lipomas are rarely encountered in children and often remain asymptomatic until they reach a significant size or complications develop. Imaging modalities such as CT and MRI play a crucial role in accurate diagnosis and preoperative planning. Complete surgical excision is curative and typically associated with excellent outcomes.

**Conclusion:**

Mesenteric lipomas are rare in children and can pose a diagnostic challenge due to their uncommon occurrence and variable clinical presentation. Vigilance during imaging, even in trauma cases, is essential for early detection. Complete surgical excision ensures excellent outcomes with minimal risk of recurrence.

## Highlights

• Mesenteric lipomas are rare benign tumors, particularly in the pediatric population.
• Clinical presentation is highly variable.
• Imaging, especially CT, is essential for accurate diagnosis and surgical planning.
• Complete surgical excision offers an excellent prognosis with a low risk of recurrence.

## 1 Introduction

Lipomas are common benign tumors composed of mature adipose tissue and can arise wherever fat is present in the body [[1], [2], [3], [4], [5]]. They are among the most frequently encountered mesenchymal neoplasms, with an estimated prevalence of approximately 1 % in the general population and an annual incidence of about 2.1 per 1000 individuals. Lipomas most commonly affect adults between 40 and 60 years of age and are typically located in superficial areas such as the trunk scalp, neck, shoulders, and limbs [4,5].

In contrast, deep-seated lipomas are less common and have been reported in locations including the thorax, intracranial, mediastinal, pelvis, intraperitoneal, retroperitoneal, and paratesticular region [5]. Mesenteric lipomas, arising from adipose tissue within the intestinal mesentery, are exceedingly rare particularly in the pediatric population with only a limited number of cases described in the literature [1,6,7].

Clinically, mesenteric lipomas are usually slow-growing, soft, and mobile. Symptoms vary depending on the size and anatomical location of the tumor. Large lesions can cause abdominal pain, distension, constipation, or even bowel obstruction or volvulus [[7], [8], [9]]. Imaging plays a critical role in diagnosis. Ultrasound, computed tomography (CT), and magnetic resonance imaging (MRI) are effective in characterizing these lesions and differentiating them from other intra-abdominal masses [10].

Although mesenteric lipomas are benign, surgical excision is indicated for symptomatic or enlarging lesions. Bowel resection may be necessary if the mass causes obstruction or involves the bowel wall. Prognosis after complete excision is excellent, with minimal risk of recurrence [9,11].

This case report aims to contribute to the limited literature on mesenteric lipomas in the pediatric population by presenting a rare case discovered incidentally during the evaluation of a child following minor trauma. The uniqueness of this case lies in the asymptomatic nature of the lesion and its unexpected detection through routine imaging performed as part of trauma assessment. It underscores the importance of maintaining a broad differential diagnosis when intra-abdominal masses are encountered and highlights the role of imaging in the incidental identification of clinically silent but potentially significant pathologies. This case report is reported in line with the SCARE checklist [12].

## 2 Case presentation

A 12-year-old female presented to our emergency department following a minor road traffic accident, during which she sustained a superficial laceration to the left leg. On examination, the patient was hemodynamically stable with no signs of systemic compromise. A plain radiograph of the affected limb excluded any underlying fracture.

**Fig. 1:**
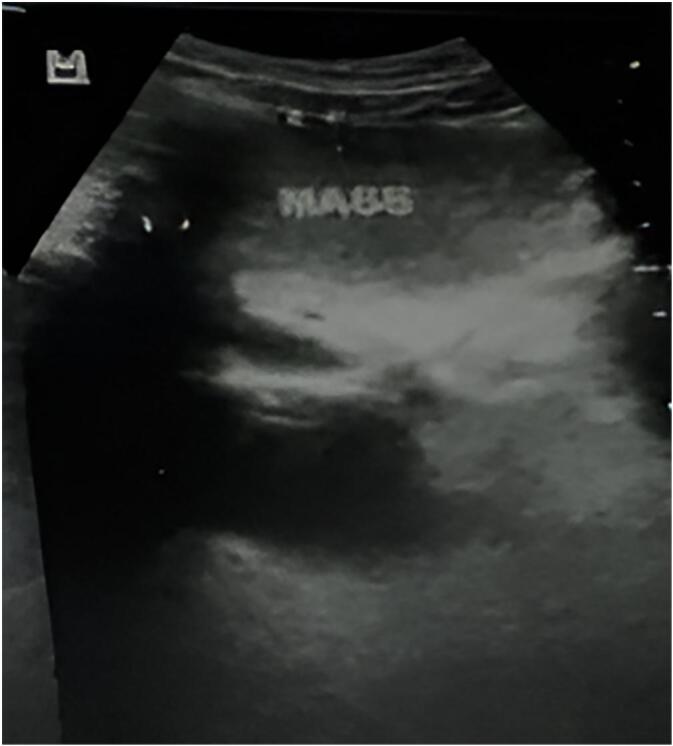
Representative sonographic image showing a well-defined, homogeneously hyperechoic intra-abdominal mass, consistent with a lipoma.

**Fig. 2:**
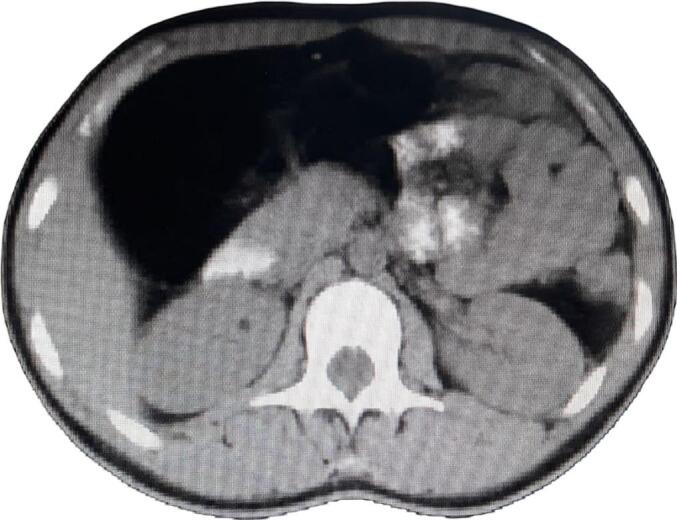
Selected cut from a contrast-enhanced (CT) scan of the abdomen revealed a well-defined, intraperitoneal, homogeneous, hypodense, non-enhancing mass located in the right upper and mid-abdomen, measuring 13.2 × 8.8 × 10.3 cm.

As part of the routine trauma evaluation, an abdominal ultrasound was performed. While no intra-abdominal injuries or free fluid were identified, abdominal ultrasound incidentally revealed a well-defined, encapsulated, homogeneous, hyperechoic intra-abdominal mass in the right upper quadrant. The lesion lacked calcifications and demonstrated minimal to no internal vascularity on Doppler imaging (Fig. 1). Further characterization with contrast-enhanced CT of the abdomen revealed a well-circumscribed, intraperitoneal, homogeneous, hypodense, non-enhancing mass located in the right upper and mid-abdomen, measuring 13.2 × 8.8 × 10.3 cm. The lesion exhibited fat attenuation values, consistent with a lipomatous tumor. It was seen displacing adjacent bowel loops without evidence of wall thickening, obstruction, or infiltration of surrounding structures. There was no evidence of bowel, biliary, or urinary tract obstruction, vascular invasion, lymphadenopathy, or ascites. The mass was closely related to the hepatic flexure of the colon; however, due to its large size and displacement of adjacent structures, the precise site of origin could not be definitively determined on imaging. The features are most consistent with a benign lipoma (Fig. 2).

Baseline laboratory investigations, including full blood count, renal function tests, and urine dipstick analysis, were all within normal limits. The patient's leg wound was managed with dressing and analgesia.

**Fig. 3:**
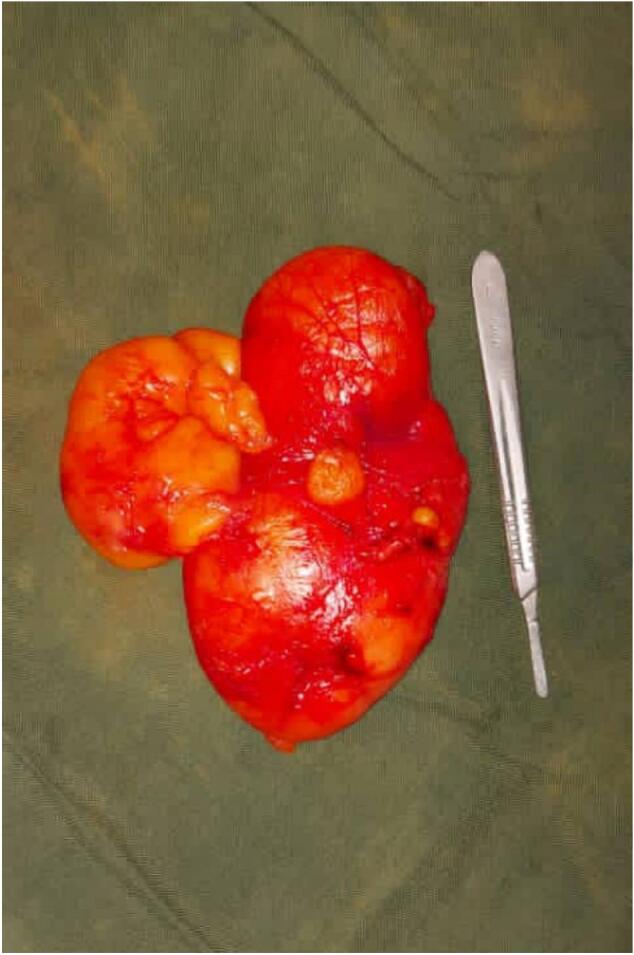
Gross specimen of the excised tumor demonstrating a large, lobulated, well-encapsulated yellowish mass consistent with a lipomatous tumor.

An elective laparotomy was subsequently performed. Intraoperatively, a solitary, encapsulated, yellowish fatty mass was identified arising from the mesentery of the transverse colon the tumor was completely excised without the need for bowel resection (Fig. 3). The postoperative course was uneventful. The patient was maintained on analgesia, tolerated oral intake after 24 h, and was discharged in stable condition 48 h postoperatively.

Histopathological examination revealed a well-encapsulated mass composed entirely of mature adipocytes. The adipocytes were uniform in size and shape, displaying small, peripherally located nuclei and abundant clear cytoplasm consistent with adipose tissue. There was no evidence of lipoblasts, cellular atypia, mitotic activity, necrosis, or infiltrative growth. The stroma was scant, consisting of delicate fibrous septa, with no signs of inflammation, hemorrhage, or vascular proliferation. No areas of myxoid change, spindle cell proliferation, or other mesenchymal elements were observed. These features are consistent with a diagnosis of a benign mature mesenteric lipoma. The patient has been followed regularly for one year postoperatively, with no evidence of recurrence.

## 3 Discussion

Mesenteric lipomas are exceptionally rare in children and are typically asymptomatic until they reach a size large enough to produce symptoms due to mass effect [1,2,8,11]. This case is unusual in that the tumor was discovered incidentally during imaging performed for unrelated trauma, and the patient was entirely asymptomatic.

A recent literature review by Zovko et al. identified only 26 pediatric cases of mesenteric lipomas. The majority of these cases presented with symptoms such as abdominal pain, distension, or vomiting, and none were discovered incidentally. The ileal mesentery was reported as the most commonly involved site [1]. In contrast, the lipoma in our patient was an incidental finding, with no associated abdominal symptoms, and originated from the transverse colon mesentery, an uncommon location rarely reported in the literature.

Farkas et al. reported 147 symptomatic small bowel lipomas involving the jejunum and ileum, with only 9 pediatric patients included. Notably, 91.2 % presented as emergencies, and abdominal pain was the predominant symptom in 75.5 % of cases. The majority of lesions were located in the ileum (59.9 %), followed by the jejunum (32 %), and only (4.8 %) are originating from the mesentery [7]. Our patient's case diverges from these patterns, reinforcing the variability of presentation and emphasizing the need to consider such lesions in differential diagnoses even in asymptomatic children.

Imaging is central to diagnosis. Ultrasound may detect abdominal lipomas as echogenic masses; however, variable patterns, especially in small lesions, can lead to misdiagnosis. CT and MRI offer greater accuracy by demonstrating typical fat attenuation (−80 to −120 HU) and clearly defining tumor origin and anatomical relationships. Thin fibrous septa and occasional calcifications may also be present. MRI provides superior tissue characterization, with lipomas exhibiting a homogeneous fat signal on all sequences [10]. Other differential diagnoses for fat-containing intra-abdominal masses in children besides lipoma include lipoblastoma, lymphangioma, liposarcoma, and teratoma, all of which were excluded in our case based on their distinctive imaging features [2]. CT was utilized in our patient due to its availability and the urgency of the case; it enabled a confident preoperative diagnosis and guided surgical planning.

Complete surgical excision remains the standard treatment for mesenteric lipomas. Although laparotomy is the most commonly used approach, laparoscopic excision has been reported in selected cases [1,7,13]. Bowel resection is reserved for patients with significant bowel involvement or complications such as ischemia or obstruction [14,15]. In our case, laparotomy was preferred due to the tumor's large size and proximity to mesenteric vessels. The tumor was successfully excised without bowel resection, demonstrating that bowel preservation is achievable in uncomplicated mesenteric lipomas.

The prognosis of mesenteric lipomas is excellent because they are benign tumors, do not infiltrate the surrounding structures, and carry no potential for malignant transformation. Recurrence is low, especially if the tumor is excised completely with an intact capsule [[1], [2], [3], [4]].

## 4 Conclusion

Mesenteric lipomas are rare in the pediatric population and can pose a diagnostic challenge due to their uncommon occurrence and variable clinical presentation. This case highlights the importance of maintaining a high index of suspicion during routine imaging, even in trauma settings, as incidental findings may uncover significant but silent pathologies. Early detection and complete surgical excision offer excellent outcomes with a minimal risk of recurrence.

## Author contribution

Abdelrahman S. Elnour: Designed the study concept, treated the patient, collected data, and wrote the manuscript.

Alshazly Mohamed, Adel M. Osman and Mosab I. Ahmed: Provided patient treatment and contributed to manuscript revision.

## Consent

Informed consent was obtained from the patients' parent for publication and any accompanying images. A copy of the written consent is available for review by the Editor-in-Chief of this journal on request.

## Ethical approval

Ethical approval to publish this case report was not applicable in our institution (Nyala Teaching Hospital, Sudan), because this report does not contain any personal information that could lead to identification of the patients.

## Guarantor

Abdelrahman S. Elnour.

## Research registration number

Not applicable.

## Funding

None.

## Conflict of interest statement

None.
